# Supplementary material for: Protein Disulphide Isomerase and NADPH Oxidase 1 Cooperate to Control Platelet Function and Are Associated with Cardiometabolic Disease Risk Factors
Source: Antioxidants (Basel). 2021 Mar 23;10(3):497. doi: 10.3390/antiox10030497 (PMC8004975; doi:10.3390/antiox10030497)
Supplement: Supplementary file 1 [file antioxidants-10-00497-s001.pdf]

SUPPLEMENTARY FIGURES

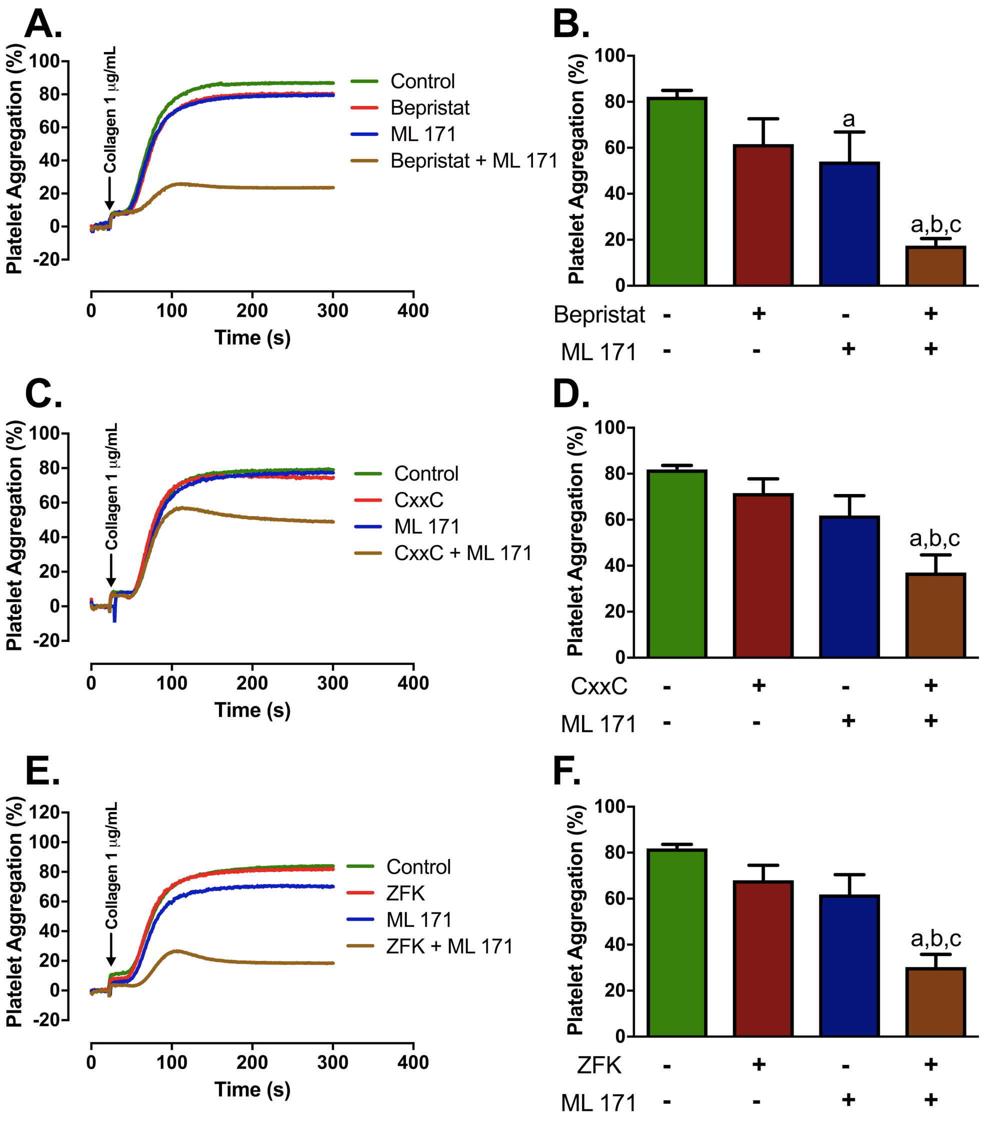

Supplementary Figure 1. Different PDI inhibitors exert similar additive inhibitory effect to ML171 in turbidimetry platelet aggregation induced by Collagen. Human WP at 4 x 10^8 platelets/mL were incubated with 0.75 µM ML171 and/or: 15 µM Bepristat (A) and (B), 50 µM CxxC peptide (C) and (D) or 1.25 µM Zafirlukast for 10 minutes, then stimulated with 1µg/mL Collagen. Aggregation traces were recorded for up to 5 minutes. Representative aggregation curves are provided in (A), (C) and (E) with corresponding summary statistics in (B), (D) and (F). n=3-5 different donors. Data on graphs show mean ± SEM. Data analysed by paired one-way ANOVA with Tukey's post-test. a $p<0.05$ *vs* first column; b $p<0.05$ *vs* second column and c $p<0.05$ *vs* third column of corresponding graph.

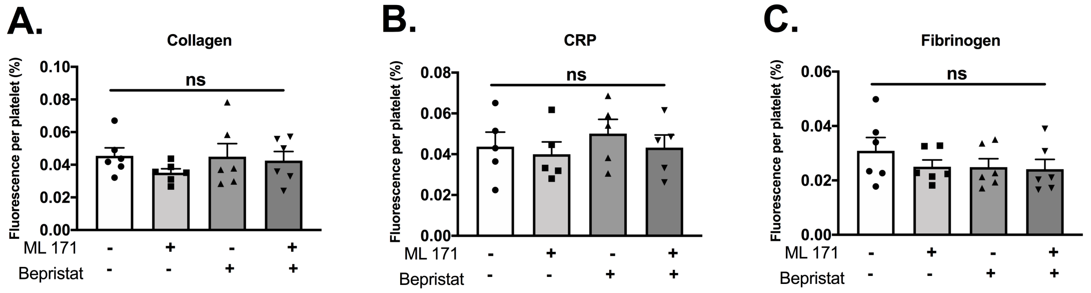

Supplementary Figure 2. No additive inhibitory effect of bepristat and ML171 on human platelet spreading. Human WP at 2 x 10^7 platelets/mL were incubated with ML171 (3 μM) and/or bepristat (15 μM) for 10 minutes and left to adhere and spread on Collagen- (A), CRP- (B) or Fibrinogen-coated (C) surfaces for 45 minutes. Platelets were labeled with fluorescently tagged phalloidin and visualized in a Nikon A1-R confocal microscope. Number of adhered platelets was divided by total fluorescence of field to obtain fluorescence per platelet as a surrogate for platelet spreading. Data on graphs show mean ± SEM. Data analysed by paired one-way ANOVA with Tukey's post-test. ns: $p>0.05$.

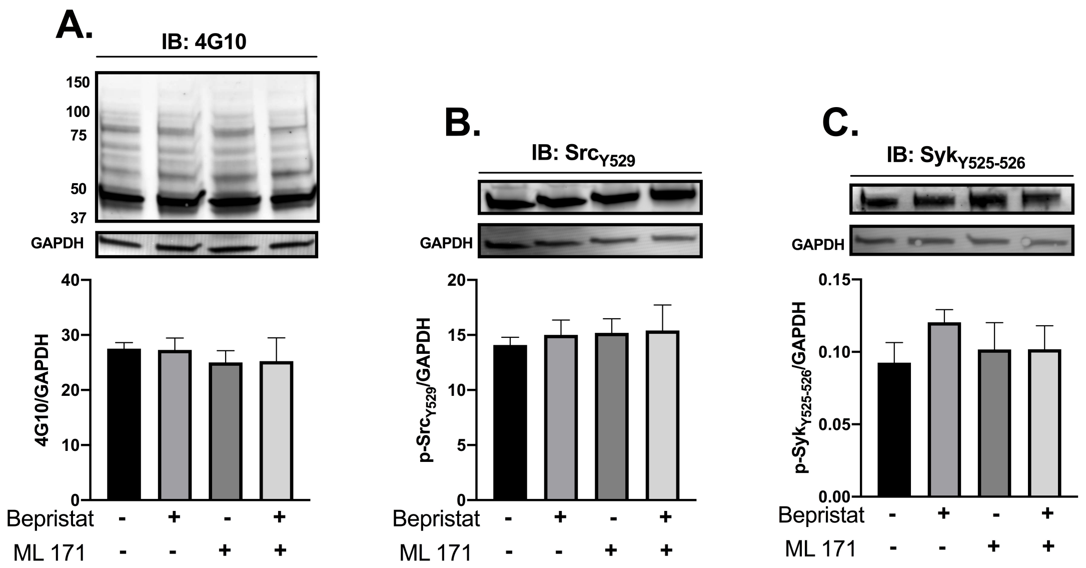

Supplementary Figure 3. PDI and Nox-1 co-inhibition did not affect tyrosine phosphorylation or phosphorylation of upstream GPVI proteins. WP at 4 x 10^8 platelets/mL were incubated with 3 μM ML171 and/or 15 μM bepristat for 10 minutes prior to the addition of 3 μg/mL Collagen. Platelets were lysed after 90 seconds and immunoblots performed. Samples were tested for: 4G10 total Tyr phosphorylation (A), Src Y529 (B) and Syk Y525-526 (C). Representative blot is presented on top of bar graphs with summary statistics. Each lane represents the condition in graph below. n=3-4 donors. Data on graphs show mean ± SEM and analysed by paired one-way ANOVA and Tukey's post-test.

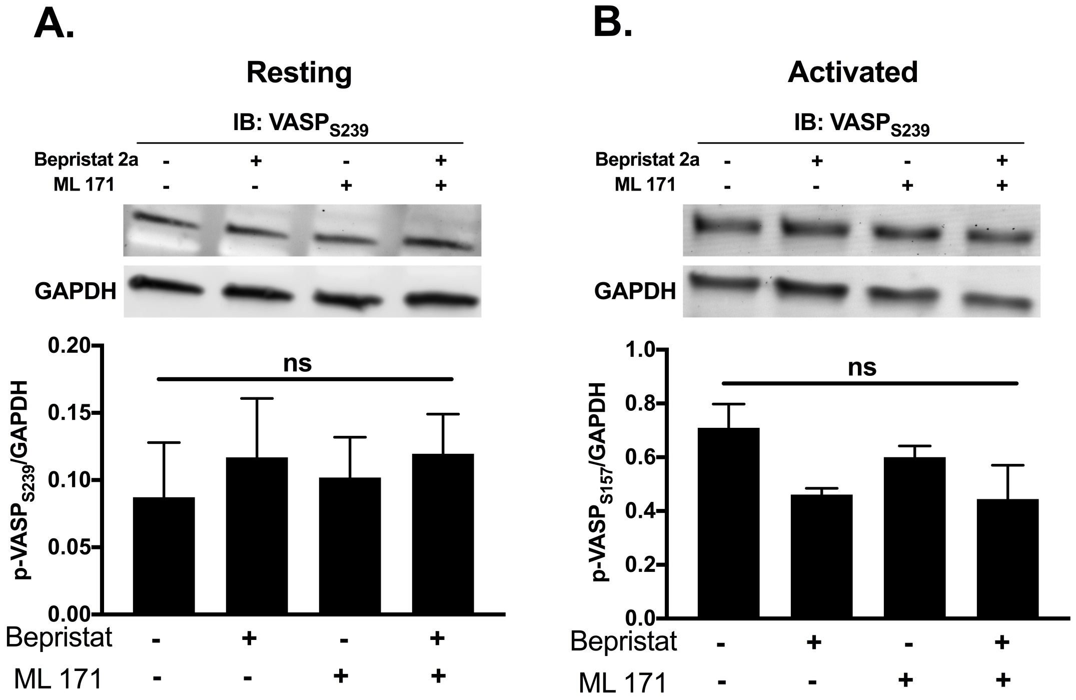

Supplementary Figure 4. VASP phosphorylation in resting and activated platelets was not affected by PDI and Nox-1 inhibition. WP at 4 x 10^8 platelets/mL were incubated with 3 µM ML171 and/or 15 µM bepristat for 10 minutes prior to the addition of 3 µg/mL collagen. Some collagen-stimulated samples were treated with vehicle alone. Platelets were lysed after 90 seconds and immunoblots performed. Samples were tested for VASP S239 phosphorylation. Representative blot is presented on top of bar graphs with summary statistics. Each lane represents the condition in graph below. n=3-4 donors. Data on graphs show mean ± SEM and analysed by paired one-way ANOVA and Tukey's post-test.

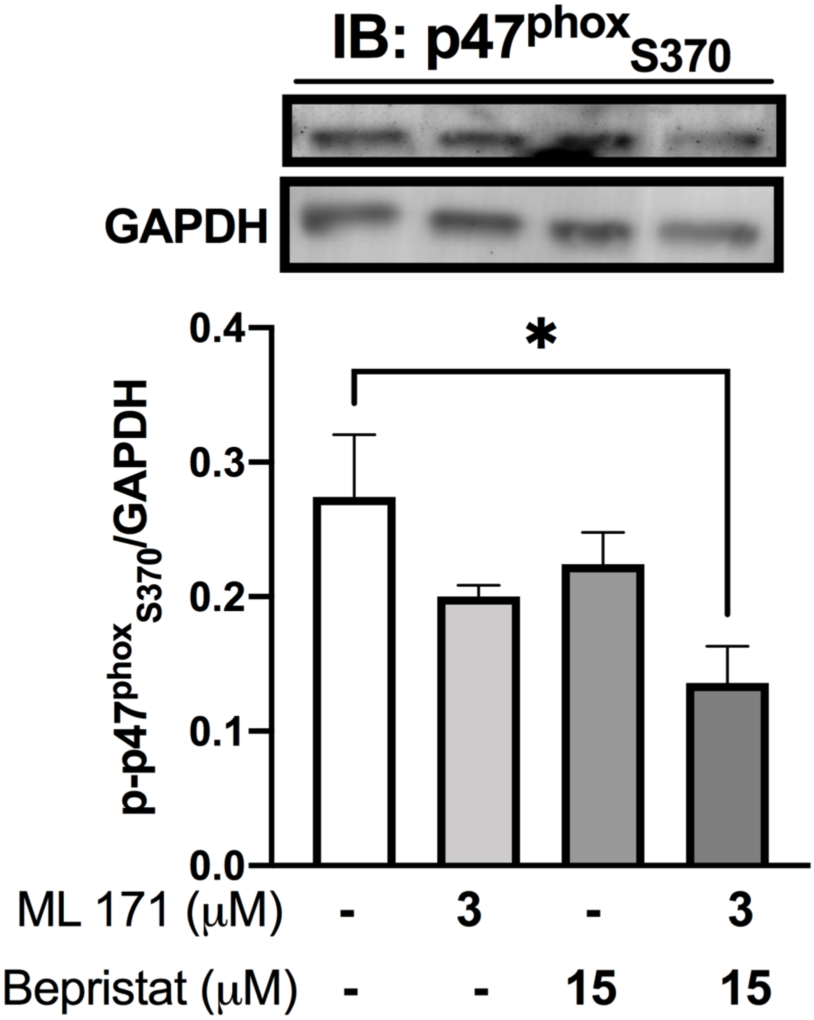

Supplementary Figure 5. PDI and Nox-1 co-inhibition decreases phosphorylation of p47phox. WP at 4 x 10^8 platelets/mL were incubated with 3 μM ML171 and/or 15 μM bepristat for 10 minutes prior to adding 3 μg/mL Collagen. Platelets were lysed after 90 seconds and immunoblots performed. Samples were tested for phosphorylation of p47phoxS370. GAPDH was used as a control for equal loading. Representative blot is presented above of bar graph with summary statistics, following normalisation for protein loading. Each lane represents the condition in graph below. Data are representative of 3-4 independent experiments. Bar graph shows mean ± SEM and was analysed by paired one-way ANOVA and Tukey's post-test. * $p<0.05$.

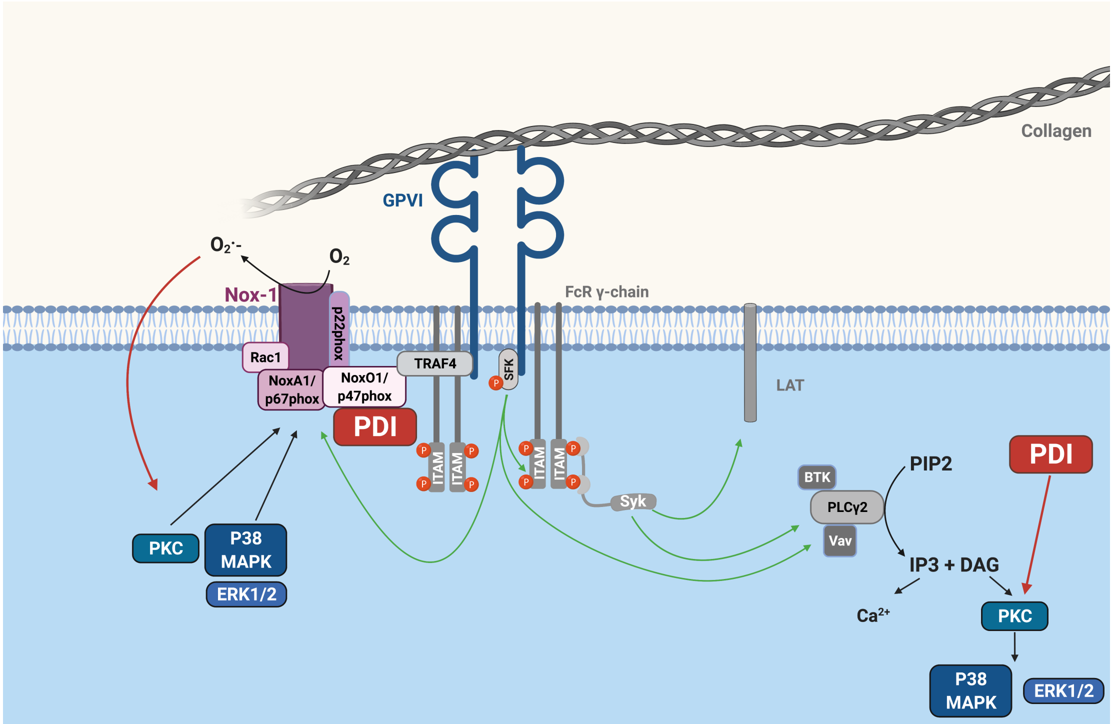

Supplementary Figure 6. Summary of molecular processes regulated by PDI and Nox-1 in collagen-mediated signal. Upon collagen binding to clustered or dimeric glycoprotein VI (GPVI), the cytosolic tail of GPVI activates Src family kinases (SFK) which phosphorylate the immunoreceptor tyrosine-based activation motif (ITAM) part of the Fc receptor γ-chain, Bruton's tyrosine kinase (BTK), and lead to activation of the Nox-1 complex that is attached to GPVI through TNF receptor-associated factor 4 (TRAF4). PDI localizes with p47phox upon activation with CRP. BTK will phosphorylate phospholipase C (PLCγ2). ITAM phosphorylation results in activation of Syk, which will phosphorylate linker for activation of T cells (LAT) protein, as well as PLCγ2, Vav and BTK. PLCγ2 will catalyse the formation of trisphosphate inositol (IP3) and diacylglycerol (DAG) from phosphatidylinositol 4,5-bisphosphate (PIP2). PIP2 may be converted to phosphatidylinositol-3,4,5-trisphosphate (PIP3) by phosphoinositide 3-kinase (PI3K). IP3 and DAG will increase intracellular Ca2+ and induce protein kinase C (PKC) activation, which will phosphorylate mitogen-activated protein kinases (MAPK). PDI regulates the activation of PKC and MAPKs, which interact with p47phox to assemble the Nox-1 complex that is responsible for superoxide generation. Superoxide may then activate PKC and MAPKs in a positive feedback loop. Green lines indicate early GPVI signalling.

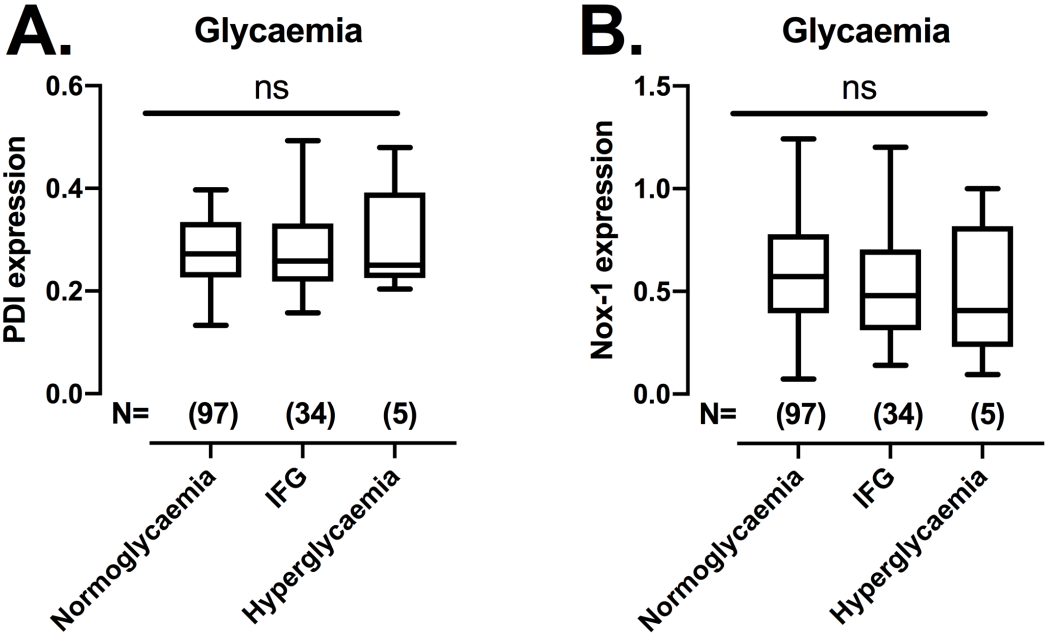

Supplementary Figure 7. Platelet PDI and Nox-1 are not increased in hyperglycaemia. Washed platelets (WP) from 136 volunteers were lysed and immunoblots performed for PDI, Nox-1 and loading control GAPDH. Anthropometric and metabolic characteristics were also collected. Value cut-offs of stratifications can be found on Supplementary Methods and were all performed according to international guidelines. PDI and Nox-1 expression were stratified by: (A and B) glycaemia in normoglycaemia (<5.6 mmol/L), impaired fasting glycaemia (IFG) (5.6 – 6.9 mmol/L) and hyperglycaemia (>6.9 mmol/L). Data in graph show box and whiskers depicting median, range and 25th and 75th percentiles analysed by one-way ANOVA and Tukey's post-test. ns: p>0.05.

SUPPLEMENTARY TABLES

Supplementary Table 1. Full blood count of WT and Nox-1$^{-/-}$ mice

	WT	Nox-1$^{-/-}$
**Red Blood Cells**		
**Hematocrit (%)**	36.67±1.368	36.00±0.806
**RBC (x10^3/µL)**	7.76±0.278	7.67±0.192
**Haemoglobin (g/dL)**	12.42±0.504	12.30±0.158
**MCV (fL)**	47.37±0.214	47.50±0.65
**MCH (pg)**	15.89±0.155	16.04±0.287
**MCHC (g/dL)**	33.54±0.336	34.20±0.626
**RDW (%)**	14.64±0.148	**15.64±0.256***
**White Blood Cells**		
**Leukocytes (x10^3 cells/µL)**	6.75±0.247	**8.22±0.407***
**Lymphocytes (x10^3 cells/µL)**	5.47±0.337	**6.82±0.296***
**Lymphocytes (%)**	82.92±1.216	82.94±0.618
**Monocytes (x10^3 cells/µL)**	0.76±0.072	0.82±0.066
**Monocytes (%)**	9.52±0.571	9.98±0.392
**Granulocytes (x10^3 cells/µL)**	0.60±0.072	0.60±0.071
**Granulocytes (%)**	8.50±1.136	7.08±0.41
**Platelets**		
**Platelet count (x10^3 cells/µL)**	507.9±22.78	468.8±20.49
**MPV (fL)**	4.95±0.043	4.98±0.111

Data presented as mean ± SEM. N= 7 mice for WT and N=5 for Nox-1$^{-/-}$. Groups were compared by unpaired Student t-test. * $p<0.05$.

Supplementary Table 2. Summary statistics of study population (n=137)

Parameter	
**Age (years)**	48.08 ± 11.46
**BMI (kg/m^2)**	25.09 ± 4.15
**Gender (M%/F%)**	(32%/68%)
**Glycaemia (mmol/L)**	5.36 ± 0.70
**Systolic BP (mmHg)**	128.3 ± 15.15
**Waist (cm)**	88.18 ± 11.13
**PDI expression**	0.27 ± 0.07
**Nox-1 expression**	0.57 ± 0.27

Data presented as mean ± SD or % for gender. BMI: body mass index. BP: blood pressure. PDI: protein disulphide isomerase. Nox-1: NADPH oxidase-1.

SUPPLEMENTARY METHODS

1. Reagents

Prostacyclin (PGI_2), Bepristat 2a, Zafirlukast, Phorbol-12-myristate-13-acetate (PMA), Thrombin Receptor Activator Peptide 6 (TRAP-6), human fibrinogen and 3,3′-Dihexyloxacarbocyanine iodide ($DIOC_6$) were purchased from Sigma-Aldrich (Dorset, UK). PAPA-NONOate and ML171 (also known as 2-acetylphenothiazine or 2APT) was purchased from Tocris (Abingdon, UK). PE/Cy5 anti human CD62P antibody was purchased from BD Biosciences (Wokingham, UK). FITC-conjugated fibrinogen was purchased from Agilent (Stockport, UK). PDI inhibitor CxxC peptide [1] was purchased from EZBiolab (Parsippany, USA). GFOGER was purchased from CambCol (Cambridge, UK). Collagen was purchased from Nycomed (Munich, Germany) whereas Collagen-Related Peptide (CRP) was obtained from Prof Richard Farndale (University of Cambridge, Cambridge, UK). Anti-PDI (NB600-1164, clone RL77), Anti-Nox-1 (NBP1-31546) were from Novus Biologicals (Bio-techne R&D Systems Europe Ltd, Abingdon, UK). Anti-p47phox, anti-phospho p47phox Ser370, 4G10 total phospho Tyr, Fura-2 AM calcium dye, Alexa-488, Alexa-568 and Alexa-647-conjugated secondary antibodies were bought from ThermoFisher (Paisley, UK). Anti-ERK1/2 and anti-p38 antibodies were purchased from Santacruz Biotechnology (Heidelberg, Germany). Anti-Akt, anti-phospho Src Tyr529, anti-phospho Syk Tyr525/526, anti-phospho vasodilator-stimulated phospho-protein (VASP) (Ser239), PKC substrate, anti-phospho-Akt Ser473, anti-phospho p38 Thr180/Tyr182, anti-phospho ERK Thr202/Tyr204 were purchased from Cell Signalling (Hitchin, UK). Anti-glyceraldehyde 3-phosphate dehydrogenase (GAPDH) was purchased from Proteintech (Manchester, UK).

2. Washed platelets preparation

Blood was collected from healthy adult volunteers who were not using antiplatelet medication and had previously provided informed consent. Platelet-rich plasma (PRP) was obtained after centrifuging whole blood at 100 x *g*, 20 minutes, 22°C. To obtain washed platelets (WP), PRP was centrifuged twice at 1000 x *g*, 10 min, 22°C in the presence of 1.25 µg/mL prostacyclin and 1:5 v/v acid citrate dextrose (ACD: 5% sodium citrate, 2% D-glucose and 1.5% citric acid). The final platelet pellet was resuspended in modified Tyrode's-HEPES buffer, (134 mM NaCl, 20 mM N-2-hydroxyethylpiperazine-N′-2-ethanesulfonic acid, 12 mM $NaHCO_3$ 5

mM glucose, 0.34 mM Na_2HPO_4, 9 mM KCl and 1 mM $MgCl_2$, pH 7.3) and rested for 30 minutes at 30 °C before experiments. The Research Ethics Committee from the University of Reading approved all protocols to obtain and use human blood samples.

3. Collection of mouse blood and platelet preparation

Colonies of Nox-1$^{-/-}$ mice were purchased from Jackson Laboratory (Sacramento, CA, USA) and C57BL/6 were used as controls, as recommended by the animal provider. Animals were kept under a 12 h light cycle, controlled temperature (22-24°C) and food and water *ad libitum*. The University of Reading Local Ethics Review Panel approved all protocols within a license from the British Home Office. Mice (11 – 14 weeks, females) were culled in a CO_2–filled chamber and blood collected through cardiac puncture in a syringe containing 3.2% sodium citrate at a 1:9 v/v citrate-blood ratio. Whole blood was centrifuged at 203 x *g* for 8 minutes and PRP collected. 1.25 µg/mL PGI_2 was added and PRP centrifuged at 1,028 x *g* for 5 min and pellet resuspended in modified Tyrode's-HEPES buffer to obtain WP.

4. Immunofluorescence microscopy

Human PRP were activated with 1 µg/mL CRP for 3 minutes in the presence of integrillin at 4 µg/mL. PRP was fixed immediately using 5% paraformaldehyde and centrifuged at 1000 x *g* for 10 minutes. The pellet was resuspended in 1:9 v/v ACD-phosphate buffer solution (PBS) and submitted to another centrifugation under the same conditions. The final pellet was resuspended in PBS containing 1% w/v BSA and left to adhere onto poly-L-lysine coverslips for 90 minutes at 37 °C. Coverslips were washed three times with PBS and blocked again in PBS containing 1% w/v BSA for 1 hour. Blocking buffer was washed away with PBS and primary or IgG control antibodies added at 1:250 v/v dilution in PBS containing 0.2% v/v Triton-X-100 and 2% v/v donkey serum and incubated at 4 °C overnight. Antibodies were washed away three times with PBS and appropriate secondary antibodies tagged with different fluorophores added for 1 hour at room temperature. Finally, coverslips were mounted in gold anti-fade onto a coverslips and analysed with a 100 x magnification oil-immersion lens on a Nikon A1-R confocal microscope (Nikon Instruments Europe BV, Amsterdam, Netherlands).

5. Turbidimetry and plate-based platelet aggregation

Platelet aggregometry was performed by turbidimetry in a four-channel AggRam aggregometer (Helena Biosciences, Gateshead, UK), as described previously [2]. Briefly, human (4 x 10^8 platelets/mL) or mouse (2 x 10^8 platelets/mL) WP were pre-incubated with inhibitors for 10 minutes before stimulation with collagen and curves recorded for up to 300 seconds. For mouse experiments, WP were pre-incubated with inhibitors for 10 minutes and stimulation obtained with 5 μg/mL collagen. The concentrations of inhibitors used are described in appropriate figure legend. To reconstruct the curves, 0% was set when t = 10 seconds and 100% set as blank (distilled water) placed at the end of the run in each channel for at least 15 seconds.

Platelet aggregation was also assessed through an end-point, plate-based method, as described previously [3]. Briefly, human WP (4 x 10^8 platelets/mL) were added to a 96-wells half-area plate (Greiner) containing varying concentrations of PDI inhibitor Bepristat or Nox-1 inhibitor ML171 and incubated for 10 minutes. Then, agonists collagen (2 μg/mL), CRP (1 μg/mL), TRAP-6 (10 μM) or PMA (500 nM) were added and plate shaken at 1200 rpm for 5 minutes at 37°C using a plate shaker (Quantifoil Instruments). Absorbance was measured at 450 nm using a Flexstation 3 plate reader.

6. Fibrinogen binding and P-selectin exposure

Human WP (4 x 10^8 platelets/mL) were incubated with 7.5 μM Bepristat and/or 0.75 μM ML171 for 10 minutes. Platelets were activated with 1 μg/mL CRP for 10 minutes and incubated with FITC-conjugated fibrinogen or PE/Cy5-conjugated anti-human CD62P for 30 minutes. This was then diluted 25 x with Tyrodes-HEPES buffer and read using a BD Accuri C6 plus flow cytometer. Platelets were gated according to forward and size scatter and analysed using the BD Accuri software.

7. Calcium measurement

Human PRP was incubated with 2 μM Fura-2 AM for 1 hour at 30°C. PRP was centrifuged at 350 *g* for 20 minutes and WP (4 x 10^8 platelets/mL) resuspended in Tyrodes-HEPES buffer. Platelets were immediately placed in a 96-wells black plate with clear bottom and incubated with 3.75 μM Bepristat and/or 3 μM ML171 for 10 minutes and stimulated with 1 μg/mL CRP. Fluorescence was read every 5 seconds

for 5 minutes using a Flexstation 3 fluorimeter (excitation 340 and 380 and emission 510 nm). Calcium signal was derived from the ratio of the 340 and 380 excitation beams.

8. Platelet spreading

Human WP (2 x 10^7 platelets/mL) were incubated with Bepristat (7.5 to 30 μM) and/or ML171 (0.1875 to 6 μM) for 10 minutes and left to adhere to collagen (30 μg/mL), fibrinogen (30 μg/mL) or CRP (10 μg/mL)-coated surfaces (96-wells plate) for 45 minutes at 37°C. Non-adherent platelets were washed off three times with PBS. Paraformadehyde 0.2% was added for 10 minutes to fix the platelets. Triton-X 0.01% v/v was added for 5 minutes to permeabilize the cells. After three washes with PBS to remove Triton-X, platelets were stained with Alexa Fluor 488-conjugated phalloidin (1:1000 v/v) for 1 hour in the dark at room temperature and analyzed using a 20x lens on a Nikon A1-R Confocal microscope.

9. Immunoblotting

Human WP (4 x 10^8 platelets/mL) were incubated with 15 μM Bepristat and/or 3 μM ML171 for 10 minutes and stimulated with 3 μg/mL collagen. On some experiments, collagen was not added in order to assess the effects of PDI and Nox-1 inhibitors in resting platelets. For mouse experiments, WP (2 x 10^8 platelets/mL) were incubated with 7.5 μM Bepristat for 10 minutes and stimulated with 5 μg/mL collagen. Platelets were lysed in reducing Laemmli buffer (12% (w/v) Sodium Dodecyl Sulphate (SDS), 30% (v/v) glycerol, 0.15 M Tris-HCl (pH 6.8), 0.001% (w/v) Brilliant Blue R, 30% (v/v) β-mercaptoethanol) and heated for five to ten minutes. SDS-PAGE and immunoblotting were performed using standard protocols exactly as described in [2]. Specific primary phosphor-antibodies were used as described in figure legends. Mouse anti-human GAPDH was used as loading controls. Membranes were visualised using a Typhoon imaging system (GE Healthcare, Hatfield, UK). For experiments using PKC substrate and 4G10 antibodies, all phosphorylated bands were normalized to the corresponding GAPDH band.

10. Tail bleeding assay

Nox-1$^{-/-}$ or C57BL/6 wildtype (WT) mice were anesthesized through an intraperitoneal injection of ketamine (100 mg/kg) and xylazine (10 mg/kg). After

animals were fully anaesthetized, Bepristat (0.5 μL of a 100 μM solution diluted in 100 μL PBS per 25 g of animal; 50 μM *in vivo* concentration) was injected intravenously. After 5 minutes, 5 mm of the tail was amputated using a sharp blade. The bleeding tail was then placed in PBS buffer kept at 37 °C and bleeding time recorded for up to 20 minutes, after which mice were terminated.

11. Population study

This study comprised of 136 volunteers aged 30 to 65 not using chronic medications that were recruited at the University of Reading to assess physical, metabolic and platelet characteristics. Volunteers answered a questionnaire about their age, gender, amongst other questions not included in this study. A competent researcher measured the height, weight, body mass index (BMI) blood pressure (BP, measured seated with an electronic automatic sphygmomanometer) and waist and hip circumferences. Blood was taken after overnight fasting and serum glucose levels measured using standard biochemistry protocols. Platelets were washed and immunoblotting performed as above. Loading control GAPDH was used to normalize levels of PDI and Nox-1 to protein loading in each well.

Volunteers were stratified according to their BMI as healthy weight (18.5 – 24.9 kg/m^2), overweight (25 – 29 kg/m^2), class 1 obesity (30 – 34.9 kg/m^2) and class 2 obesity (35 – 39.9 kg/m^2). BP was stratified according to the International Society of hypertension [4]: normal (systolic <130 and diastolic <85 mmHg), high-normal (systolic 130-139 and/or diastolic 85-89 mmHg), grade 1 hypertension (systolic 140-159 and/or diastolic 90-99 mmHg) and grade 2 hypertension (systolic ≥160 and/or diastolic ≥100 mmHg). Glycaemia was stratified according to the American Diabetes Association [5]: normoglycaemia (<5.6 mmol/L), impaired fasting glycaemia (IFG) (5.6 – 6.9 mmol/L) and hyperglycaemia (>6.9 mmol/L). Waist circumference was stratified according to the European Society of Cardiology [6]: normal (Caucasian men <94 cm; men of other ethnicities <90 cm; women <80 cm) and central obesity (Caucasian men ≥94 cm; men of other ethnicities ≥90 cm; women ≥80 cm).

12. Statistical analysis

Statistical analyses were performed on GraphPad Prism 8.0 software (GraphPad Software, San Diego, USA). Bar graphs and tables express mean ± SEM. Sample size varied from 4-6 independent repeats for *in vitro* experiments and between

6 and 8 for tail bleeding experiments. Outliers were determined and excluded by ROUT test. For *in vitro* experiments using inhibitors, statistical analysis was performed through paired one-way ANOVA and Tukey as post-test, whereas for *in vivo* experiments using Nox-1$^{-/-}$ mice, these were analysed through two-way ANOVA and Sidak's multiple comparisons test.

For the population study, linear regression was used to assess the correlation between platelet PDI and Nox-1 levels. To assess the possible association of platelet Nox-1 and PDI with risk factors for metabolic syndrome, volunteers were stratified according to their BMI, BP, waist circumference and glycaemia. Analysis was performed through unpaired one-way ANOVA and Tukey as post-test.

REFERENCES

1. Sousa HR, Gaspar RS, Sena EM, da Silva SA, Fontelles JL, AraUjo TL, Mastrogiovanni M, Fries DM, Azevedo-Santos AP, Laurindo FR, Trostchansky A, Paes AM. Novel antiplatelet role for a protein disulfide isomerase-targeted peptide: Evidence of covalent binding to the c-terminal cghc redox motif. *J Thromb Haemost*. 2017;15:774-784
2. Gaspar RS, da Silva SA, Stapleton J, Fontelles JLL, Sousa HR, Chagas VT, Alsufyani S, Trostchansky A, Gibbins JM, Paes AMA. Myricetin, the main flavonoid in syzygium cumini leaf, is a novel inhibitor of platelet thiol isomerases pdi and erp5. *Front Pharmacol*. 2019;10:1678
3. Bye AP, Unsworth AJ, Desborough MJ, Hildyard CAT, Appleby N, Bruce D, Kriek N, Nock SH, Sage T, Hughes CE, Gibbins JM. Severe platelet dysfunction in nhl patients receiving ibrutinib is absent in patients receiving acalabrutinib. *Blood Adv*. 2017;1:2610-2623
4. Unger T, Borghi C, Charchar F, Khan NA, Poulter NR, Prabhakaran D, Ramirez A, Schlaich M, Stergiou GS, Tomaszewski M. 2020 international society of hypertension global hypertension practice guidelines. *Hypertension*. 2020;75:1334-1357
5. Association AD. 2. Classification and diagnosis of diabetes: Standards of medical care in diabetes—2019. *Diabetes care*. 2019;42:S13-S28
6. Mach F, Baigent C, Catapano AL, Koskinas KC, Casula M, Badimon L, Chapman MJ, De Backer GG, Delgado V, Ference BA. 2019 esc/eas guidelines for the management of dyslipidaemias: Lipid modification to reduce cardiovascular risk. *Atherosclerosis*. 2019;290:140-205
